# miR-622 Increases miR-30a Expression through Inhibition of Hypoxia-Inducible Factor 1α to Improve Metastasis and Chemoresistance in Human Invasive Breast Cancer Cells

**DOI:** 10.3390/cancers16030657

**Published:** 2024-02-03

**Authors:** Chun-Wen Cheng, Yu-Fan Liu, Wen-Ling Liao, Po-Ming Chen, Yueh-Tzu Hung, Huei-Jane Lee, Yu-Chun Cheng, Pei-Ei Wu, Yen-Shen Lu, Chen-Yang Shen

**Affiliations:** 1 Institute of Medicine, Chung Shan Medical University, Taichung 40201, Taiwan; yaoming9@yahoo.com.tw (P.-M.C.); acb221zz4@gmail.com (Y.-T.H.); 2 Department of Medical Research, Chung Shan Medical University Hospital, Taichung 40201, Taiwan; 3 Department of Biomedical Sciences, Chung Shan Medical University, Taichung 40201, Taiwan; yfliu@csmu.edu.tw; 4 School of Medicine, China Medical University, Taichung 40604, Taiwan; wl0129@mail.cmu.edu.tw; 5 Department of Medical Genetics and Medical Research, China Medical University Hospital, Taichung 40604, Taiwan; 6 Department of Biochemistry, School of Medicine, College of Medicine, Chung Shan Medical University, Taichung 40201, Taiwan; lhj@csmu.edu.tw; 7 Department of Internal Medicine, Cathay General Hospital, Taipei 10629, Taiwan; yuchuncheng0817@gmail.com; 8 Institute of Biomedical Sciences, Academia Sinica, Taipei 11529, Taiwan; peiei@gate.sinica.edu.tw; 9 Department of Oncology, National Taiwan University Hospital, Taipei 10022, Taiwan; yslu@ntu.edu.tw; 10 Department of Internal Medicine, National Taiwan University Hospital, Taipei 10022, Taiwan; 11 College of Public Health, China Medical University, Taichung 40604, Taiwan

**Keywords:** breast cancer, hypoxia-inducible factor 1α, epithelial–mesenchymal transition, autophagy, microRNA

## Abstract

**Simple Summary:**

Hypoxia-inducible factor 1α (HIF-1α) is a driver of tumor cell metastasis through epithelial–mesenchymal transition (EMT) in solid tumors, while autophagy increases invasiveness and drug resistance in the hypoxic environment of breast cancer. Our study demonstrates that inducing miR-622 inhibits HIF-1α, subsequently activating miR-30a expression. This orchestrated modulation resulted in a significant reduction in the invasive and migratory capabilities of MDA-MB-231 breast cancer cells, making them more responsive to docetaxel treatment. These findings underscore the therapeutic potential of miR-622-induced miR-30a in disrupting HIF-1α-mediated EMT and autophagy, offering innovative approaches to the treatment of aggressive breast cancer.

**Abstract:**

Hypoxia-inducible factor 1α (HIF-1α) plays a pivotal role in the survival, metastasis, and response to treatment of solid tumors. Autophagy serves as a mechanism for tumor cells to eliminate misfolded proteins and damaged organelles, thus promoting invasiveness, metastasis, and resistance to treatment under hypoxic conditions. MicroRNA (miRNA) research underscores the significance of these non-coding molecules in regulating cancer-related protein synthesis across diverse contexts. However, there is limited reporting on miRNA-mediated gene expression studies, especially with respect to epithelial–mesenchymal transition (EMT) and autophagy in the context of hypoxic breast cancer. Our study reveals decreased levels of miRNA-622 (miR-622) and miRNA-30a (miR-30a) in invasive breast cancer cells compared to their non-invasive counterparts. Inducing miR-622 suppresses HIF-1α protein expression, subsequently activating miR-30a transcription. This cascade results in reduced invasiveness and migration of breast cancer cells by inhibiting EMT markers, such as Snail, Slug, and vimentin. Furthermore, miR-30a negatively regulates beclin 1, ATG5, and LC3-II and inhibits Akt protein phosphorylation. Consequently, this improves the sensitivity of invasive MDA-MB-231 cells to docetaxel treatment. In conclusion, our study highlights the therapeutic potential of inducing miR-622 to promote miR-30a expression and thus disrupt HIF-1α-associated EMT and autophagy pathways. This innovative strategy presents a promising approach to the treatment of aggressive breast cancer.

## 1. Introduction

Hypoxia is a prevalent condition in solid tumors, driven by their rapid growth, insufficient blood supply, and inefficient oxygen delivery. When faced with hypoxia, there is an increase in hypoxia-inducible factor 1α (HIF-1α), an oxygen-dependent transcriptional activator. HIF-1α is responsible for the transcription of genes that allow adaptation to hypoxic environments, promoting tumor cell progression by maintaining growth, facilitating vascular mimicry and conferring properties such as invasion, migration, and drug resistance [1,2]. A growing body of evidence has deciphered how deregulation of hypoxia/Snail signaling fosters epithelial–mesenchymal transition (EMT). This transition allows epithelial cells to acquire characteristics akin to mesenchymal cells, enhancing the invasion and migration of cancer cells [3,4]. Given its pivotal role in cancer biology, HIF-1α has emerged as a promising target for diagnostic, prognostic and therapeutic purposes, providing a foundation for the development of more effective cancer treatments.

Autophagy, a cellular degradation programming based on lysosomes, plays a key role in preserving bioenergetic equilibrium by eliminating damaged cellular organelles. Initially acting as a protective response to hypoxic stress, excessive autophagy activation, through gene modulation or therapeutic interventions, can exacerbate apoptosis-induced cell death, thus limiting tumor growth [5]. In the context of cancer progression, a significant interaction has been observed between oxygen-depleted conditions and autophagy. Tumor growth often outperforms blood supply, leading to an increase in HIF-1α expression within hypoxic regions. This in turn triggers signaling pathways that facilitate tumor vascularization, ensuring a steady supply of oxygen and nutrients in the tumor microenvironment [1,6]. The central regulator of autophagy, the beclin 1 protein (encoded by the *Becn1* gene), plays a central role in the recruitment of ATG5, which in turn is indispensable for LC3 lipidation (LC3-II) during autophagosome formation [7]. Furthermore, tumor cell autophagy has been observed to be associated with HIF-1α expression, contributing to a poor prognosis and the promotion of chemoresistance in cancer [8,9]. However, the intricate interplay between hypoxia and autophagy in the context of cancer cell development remains an actively explored research area, with results influenced by genetic factors that have not yet been fully elucidated.

MicroRNAs (miRNAs) constitute a class of non-coding RNA molecules, typically composed of approximately 21–25 nucleotides, playing a pivotal role in post-transcriptional gene regulation. Through non-complementary binding to target mRNA molecules, miRNAs can either degrade the target mRNA or inhibit its translation, thus orchestrating a wide range of physiological processes [10]. In the context of tumorigenesis, specific miRNAs, called miR-ONCs, function as oncogenes, driving tumor cell growth, facilitating cancer cell invasion, and evading cell death when overexpressed. On the contrary, other miRNAs function as suppressors, preserving normal cellular processes by curbing cancer cell proliferation, invasion, and metastasis [11]. Our prior research has revealed that late-stage breast cancer cell lines exhibit reduced levels of miR-30a compared to their early-stage counterparts [12]. Clinically, we have observed that a substantial percentage of patients with advanced stages of invasive ductal carcinoma show reduced levels of miR-30a. This reduction is quantified by a more than twofold decrease in microdissected tumor cells compared to adjacent non-tumor breast cells [13]. Furthermore, miR-30a undergoes downregulation in prostate cancer cells and modulates radiosensitivity under hypoxic conditions [14]. Recent in vitro and in vivo studies in our laboratory have demonstrated that ectopically expressed miR-622 can effectively suppress lung tumor metastasis by repressing HIF-1α expression [15]. Based on this knowledge, our inference suggests that the enhanced effect of miR-622 on activating miR-30a transcriptional expression by inhibiting HIF-1α in hypoxia state may inhibit invasive behavior in breast cancer. In particular, the interaction between miR-622 and miR-30a exerts its influence on obstruction of cancer drug resistance by modulating autophagy signaling in responding hypoxic tumor environments. In this study, our findings illuminate the potential of miRNA-to-miRNA interactions as a foundational concept for the development of innovative therapeutic strategies against metastatic breast cancer.

## 2. Materials and Methods

### 2.1. Cell Culture

Human breast cancer cell lines, including Hs578T, MDA-MB-231, and MCF-7, were obtained from the American Type Culture Collection (Manassas, VA, USA). These cells were cultured in Dulbecco’s modified Eagle’s medium (DMEM, Life Technologies, Inc., Grand Island, NY, USA) or RPMI-1640 medium, each containing 0.1 mM sodium pyruvate, 10% FBS, 2 mM L-glutamine, 100 IU/mL penicillin, and 100 µg/mL streptomycin (BioSource, Rockville, MD, USA), and supplemented with 10% fetal bovine serum. For cell incubation under hypoxia-like conditions, cells were treated with deferoxamine mesylate (Sigma-Aldrich, St. Louis, MO, USA) or cultured at a constant temperature of 37 °C in a hypoxia chamber (1% O_2_, 5% CO_2_, and 94% N_2_ atmosphere).

### 2.2. Plasmid Construction and Virus Infection of Cells

We obtained the lentiviral vector pLKO (control) and pLKO/miR-622, as well as the pCMVΔR8.91 (packaging plasmid) and the envelope plasmid pMDG, from the National RNAi Core Facility at Academic Sinica, Taipei, Taiwan. The nucleotide sequences of the primer set designed to construct pri-miR-622 are: forward, 5′-CCCAAGCTTGGCTTACAAGCCCAGATTGA-3′ and reverse, 5′-CCCGAATTCCAAGCTGGCCTTCAGATTTC-3′. To generate lentivirus, 2 × 10^6^ HEK293T cells (human embryonic kidney) were transfected with 10 μg of the lentivirus-based expression vector pLKO (control) or pLKO/miR-622. At 24 h after transfection, virus-containing supernatants were collected. Subsequently, the virus-containing solution was introduced into breast cancer cells (1 × 10^6^) at the desired multiplicity of infection in the presence of 8 ng/mL polybrene for lentiviral infection. After 48 h, we selected stable transfectants under 5 µg/mL puromycin. The level of expression of miRNA was verified by real-time PCR.

### 2.3. Real-Time PCR with Reverse Transcription

Total RNA was extracted from frozen cultured cells using TRIzol reagent (Invitrogen, Carlsbad, CA, USA) following the manufacturer’s instructions. The concentration and purity of RNA were assessed using a NanoDrop ND-1000 spectrophotometer (NanoDrop Technologies, Wilmington, DE, USA). Mature miRNA levels were quantified using an NCode^TM^ miRNA first-strand cDNA synthesis kit, and a qRT-PCR kit (Invitrogen, Carlsbad, CA, USA). U6 snRNA (RNU6B) served as an endogenous control. Specific probes and primer sets were used to detect the expression of genes related to hsa-miR-622 (AB assay ID: 001553) and hsa-miR-30a (AB assay ID: 000417). All measurements were replicated in at least three independent experiments. The 2^−ΔΔCt^ method was applied to calculate relative transcript levels.

### 2.4. Cell Viability and Cell Cycle Distribution

Cell viability was assessed using the methyl thiazolyl tetrazolium (MTT) assay. For the assessment of cell cycle distribution, cells transfected with pLKO/miR-622 or the control were subjected to flow cytometric analysis. After collection, the proportions of cells in the sub-G1, G0/G1, S, and G2/M phases were analyzed using a FACSCalibur cell sorter. The resulting data were processed using CellQuest Pro software (V5.2.2) from Becton Dickinson, Biosciences (Franklin Lakes, NJ, USA).

### 2.5. Invasion and Migration Assays

A quantitative analysis of tumor cell invasion and migration abilities was performed using Boyden chamber and transwell migration assays. Cells were first trypsinized and collected from culture plates through a brief centrifugation. Subsequently, 5 × 10^4^ cells were seeded in modified Boyden chambers (Neuro Probe, Cabin John, MD, USA) equipped with polycarbonate membranes with an 8 μm pore size. Cell migration and invasion were assessed over a 16 h period, with migration performed without Matrigel and invasion performed with Matrigel. Subsequently, the cells that invaded the lower membrane surface were fixed with methanol and stained with Giemsa solution (Sigma-Aldrich Co., St. Louis, MO, USA). Quantification of invaded cells was carried out by counting five random high-power fields, and this analysis was performed using an Olympus Ckx41 light microscope (Tokyo, Japan).

### 2.6. Total Protein Extraction and Western Blotting Analysis

Whole-cell extracts were prepared and subjected to Western blotting following a protocol described previously [12]. Cell lysis involved a buffer composed of 50 mM Tris-HCl mM (pH 7.4), 100 mM NaCl, 5 mM EDTA, 50 mM NaF, 10 mM NaPP, and 1% Triton X-100, supplemented with 1 mM DTT and a cocktail of protease inhibitors from Roche Applied Science (Indianapolis, IN, USA). The total protein concentration was determined by the Bradford assay, employing standard procedures that ensure comparability among the quantities of the lysate. The samples loaded onto SDS–polyacrylamide gels were adjusted to a final amount of 30 μg/lane using appropriate volumes of stock sample, along with the reducing agent, the lysis buffer, and the tracking sample buffer. The proteins were subsequently transferred onto an Immobilon-PSQ polyvinylidene difluoride (PVDF) membrane. Following transfer, the membrane was incubated with a diluted primary antibody in 5% skim milk powder in PBST (phosphate-buffered saline and Tween-20) buffer for approximately 18 h at 4 °C. Primary antibodies targeting specific proteins were used: human HIF-1α (66730-1-Ig) and beclin 1 (11306-1-AP) from Proteintech Group, Inc. (Chicago, IL, USA); Slug (9585) and vimentin (5741) from Cell Signaling Technology (Danvers, MA, USA); caspase 9 (7885), Snail (10432), phosphorylated AKT (16646-R), and phosphorylated ERK (7383) from Santa Cruz Biotechnology (Dallas, TX, USA); and p53 (128135), ATG5 (31372), and LC3B (127375) from GeneTex International Co., Ltd. (Hsinchu City, Taiwan). For normalization of protein levels, an antibody against β-actin obtained from Sigma-Aldrich (St. Louis, MO, USA) was used as an endogenous control. Horseradish peroxidase-conjugated goat anti-mouse IgG antibody (Jackson ImmunoResearch Inc., West Grove, PA, USA) served as the second antibody, and signal detection was achieved using the Immoblon Western Chemiluminescent HRP Substrate (Millipore Corporation, Temecula, CA, USA). The PVDF membranes were detected by chemiluminescence and imaging was performed using the LAS-4000 system with the Fujifilm Image Read LAS-4000 software version 2.0. ImageJ software (version 1.52; National Institute of Health, Bethesda, MD, USA) was used to quantify the protein band intensities of the immunoblots.

### 2.7. Cellular Transfection and Luciferase Reporter Assay

The DNA sequence of the miR-30a promoter, located on chromosome 6q12–13, was obtained from the NCBI website (https://www.ncbi.nlm.nih.gov/nuccore/6522967 (accessed on 24 January 2013)). Three fragments located upstream of the 5′-UTR of the pri-miR-30a gene, denoted −1453/−1, −3142/−1328, and −5032/−3041, were extracted from the genomic DNA of MDA-MB-231 cells and subsequently integrated into the pGL4.21-Basic-Luc vector (Promega Co., Madison, WI, USA). PCR amplification was carried out using the following primer sets: −1453/−1: forward, 5′-AGCTCTCGAGTAACTTAAAAGAAGTGGGTAT-3′ and reverse, 5′-AGCTTTCGAAGTCGCTCACTGTCAACAGCAAT-3′; −3142/−1328: forward, 5′-AGCTCTCGAGAAGGGAAGGTTCATCATTA-3′ and reverse, 5′-AGCTTTCGAAGTGCTGTGCAATTCTACAT-3′; −5032/−3041: forward, 5′-GTACGGTACCGGTACTGTCTACATTACTCT-3′ and reverse, 5′-TCGAGAGCTCTGATTCATTCTTTTACACATAC-3′. The luciferase activity assay was performed according to the manufacturer’s protocol provided by the Luciferase Assay System (Promega Co., Madison, WI, USA). Briefly, cells (1 × 10^4^) were cultured in 12-well plates and 1 µg of promoter reporter construct, along with 1 µg of the pRL Renilla luciferase vector (used as a negative control) were transfected into cells using Lipofectamine 2000 reagent (Thermo Fisher Scientific, Inc., Waltham, MA, USA). Following a 24 h incubation, cells were lysed and cell lysates were collected to detect firefly luciferase and Renilla luciferase using a commercial kit as described above. The ratio of firefly luciferase to Renilla luciferase activity was measured in triplicate in three independent experiments.

### 2.8. Chromatin Immunoprecipitation (ChIP) Assay

Cell chromatin was sonicated and immunoprecipitated using a rabbit polyclonal antibody against HIF-1α or rabbit IgG as a negative control. Chemical cross-links were reversed by overnight incubation at 65 °C in the presence of 8 M NaCl. Subsequently, proteinase K (10 mg/mL) was added for 1 h at 45 °C and RNase (10 mg/mL) for 30 min at 37 °C. After extraction and precipitation, the DNA was dissolved in 30 µL of ddH_2_O. Specific regions of the miR-30a promoter, covering 181 base pairs containing and containing the predicted HIF-1α-binding sites, were amplified using the following primer set: forward, 5′-CAGATGCCAGACAATTTTACCAG-3′ and reverse, 5′-GTAAATACTAAGCAAACAACCTC-3′. The resulting PCR products were analyzed by electrophoresis on 2% agarose gels and visualized with ethidium bromide staining.

### 2.9. Flow Cytometer Determination of Apoptosis

Detection of apoptosis followed the protocols outlined in the Alexa Fluor^®^ 488 Annexin V/Dead Cell Apoptosis Kit (Thermo Fisher Scientific, Inc., Waltham, MA, USA) with slight modifications. Annexin V–PI staining was carried out by flow cytometry after 48 h of docetaxel treatment. The FACSCalibur instrument was used to collect a minimum of 10,000 events. Data analysis involved gating cell populations based on scatterplots of PI (*y*-axis) versus annexin V (*x*-axis). Cells positive for annexin V alone (lower-right quadrant, early apoptosis) and those positive for both annexin V and PI (upper-right quadrant, late apoptosis) were classified as apoptotic cells. The depicted results represent one experiment selected from three independent experiments.

### 2.10. Statistical Analysis

The statistical significance of the experimental data, categorized by a single variable, was determined through the unpaired two-tailed Student’s *t*-test, a one-way analysis of variance or the Dunnett test, as appropriate for each analysis. All statistical analyzes were performed with SPSS version 19.0 (SPSS Inc., Chicago, IL, USA). The significance levels are denoted by asterisks as follows: * *p* < 0.05, ** *p* < 0.01, *** *p* < 0.001.

## 3. Results

### 3.1. Inverse Association between miR-622 and HIF-1α Expression in Breast Cancer Cell Lines

**Figure 1:**
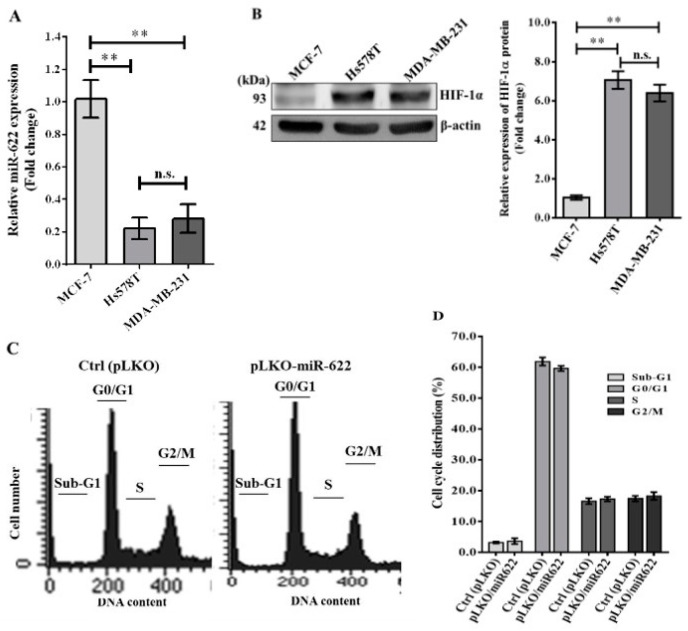
Illustration of the cell cycle analysis in breast cancer cells treated with miR-622. (**A**) Evaluation of miR-622 levels in both non-metastatic (MCF-7) and metastatic (Hs578T and MDA-MB-231) breast cancer cell lines. Quantification of the relative miR-622 levels was performed using TaqMan real-time PCR and normalized to RNU6B. (**B**) Analysis of HIF-1α protein expression by Western blotting. Quantification of HIF-1α protein levels with β-actin serving as an internal control. Data are presented as means ± S.D. (n = 3). n.s. indicates non-significant and ** denotes *p* < 0.01. (**C**) Cells were transfected with pLKO alone (control, Ctrl) or pLKO with miR-622 (pLKO-miR-622) for 48 h. Flow cytometry was used to analyze the percentage of cells at each stage of the cell cycle after DNA staining with propidium iodide. (**D**) Determination of the percentages of cells in different stages of the cell cycle. Data represent the means ± S.D. of three independent experiments. The original western blot figures can be found in File S1.

We evaluated the relative expression levels of endogenous miR-622 transcripts and HIF-1α protein in various breast cancer cell lines using qRT-PCR and Western blot techniques, respectively. In particular, a marked decrease in miR-622 expression was evident in the highly aggressive Hs578T and MDA-MB-231 breast cancer cell lines compared to the non-invasive MCF-7 cell line (Figure 1A). Concurrently, Western blot analysis confirmed an inverse correlation between miR-622 and HIF-1α expression (Figure 1B). Our observations revealed significantly elevated endogenous HIF-1α expression in both Hs578T (stage III) and MDA-MB-231 (stage IV) breast cancer cell lines compared to the MCF-7 (stage I) cell line. Previous research has proposed the presence of an miR-622 sequence complementary to the 3′-untranslated region (3′-UTR) of HIF-1α in lung cancer cells [15]. To validate this finding, we performed a dual luciferase reporter assay using a construct derived from pGL4.13. The set of primers designed for the primary miR-622 transcript was synthesized and incorporated into the lentiviral vector system following established protocols [15]. Our results unequivocally establish that miR-622 directly targets the 3′-UTR of HIF-1α mRNA in breast cancer cells (Figure S1). In addition, we investigated whether miR-622 overexpression influences cell proliferation. Analysis of cell cycle stages, including sub-G1, G0/G1, S, and G2/M phases, determined by FACS analysis with propidium iodide (PI) staining (Figure 1C), revealed no significant differences in cell proportions between MDA-MB-231 cells transfected with pLKO/miR-622 and the control group (Ctrl, vector pLKO alone) at any stage (Figure 1D).

### 3.2. MiR-622 Suppresses EMT to Decrease Metastasis of Breast Cancer Cells

**Figure 2:**
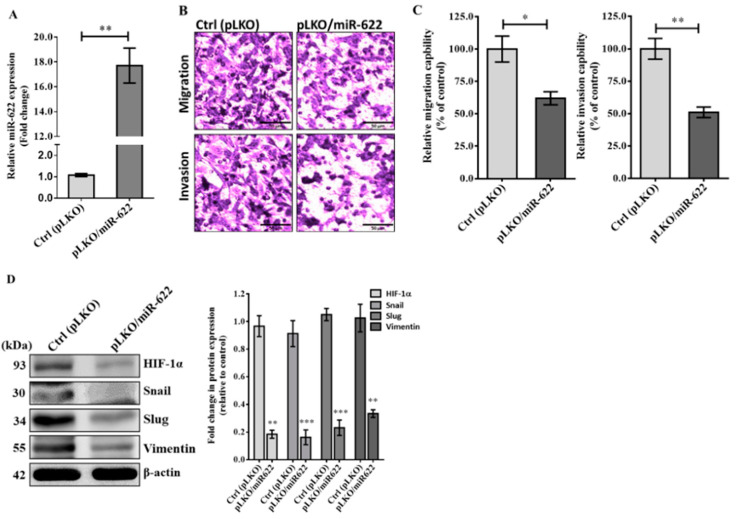
MiR-622 exerts its inhibitory effect on the invasiveness of breast cancer cells by suppressing HIF-1α to negatively regulate EMT-related proteins. (**A**) The relative miR-622 level was assessed using TaqMan real-time PCR and normalized to RNU6B. (**B**) In the Boyden chamber assay, representative micrographs show migration (**top panel**) and invasion (**bottom panel**) of MDA-MB-231 cells transfected with pLKO/miR-622 compared to the control group (pLKO alone). The scale bar represents 50 μm. (**C**) Cell counts were obtained from five random fields of view, and the quantitative analysis of migration and invasion capabilities is shown as the means ± S.D. of three independent experiments. (**D**) Western blot analysis demonstrates reduced levels of HIF-1α and EMT markers, including Snail, Slug, and vimentin, in MDA-MB-231 cells carrying miR-622 compared to the control group. Quantitative analysis of Western blot results, with β-actin was used as an internal control for protein loading. Statistical significance is denoted by asterisks: * *p* < 0.05, ** *p* < 0.01, and *** *p* < 0.001. The original western blot figures can be found in File S1.

Hypoxia is a common condition in most solid tumors, and extensive research has highlighted the crucial role of HIF-1α in the regulation of various EMT transcription factors that contribute to cancer cell progression [16,17]. Consequently, we investigated the relationship between miR-622 expression and the potential for in vitro invasion and metastasis of breast cancer. Initially, both Hs578T and MDA-MB-231 cell lines were classified as triple-negative breast cancer (TNBC) cells, characterized by the absence of estrogen receptor (ER), progesterone receptor (PR), and human epidermal growth factor receptor 2 (HER2). Furthermore, the MDA-MB-231 cell line has been extensively used to investigate the mechanisms of breast cancer metastasis. Thus, we chose the MDA-MB-231 cell line for further exploration to examine a causal link between miR-622 expression and invasiveness in breast cancer by modulating the EMT-related pathway. Using the lentivirus-based expression vector pLKO, the cell line transfected with pLKO/miR-622 exhibited an approximate 18.0-fold higher expression of miR-622 compared to the control group (pLKO) (Figure 2A). As expected, the migration and invasion capabilities of miR-622-transfected MDA-MB-231 cells were evident in the Boyden chamber assay (Figure 2B), revealing a significant reduction of more than 40% compared to mock transfected controls (Figure 2C). This observation is consistent with previously published work, which confirms the tumor-suppressive function of miR-622 in inhibiting the invasiveness and metastasis of cancer cells of various types [15,18,19]. Additional results from the Western blot assay demonstrated a significant decrease in HIF-1α level along with mesenchymal biomarkers, including Snail, Slug, and vimentin, in MDA-MB-231 cells carrying miR-622 (pLKO/miR-622) (Figure 2D). These findings indicate the mechanism underlying HIF-1α repression through miR-622, thus negatively modulating EMT to inhibit breast cancer cell metastasis.

### 3.3. Restoration of HIF-1α-Mediated EMT after Treatment with the miR-622 Inhibitor

**Figure 3:**
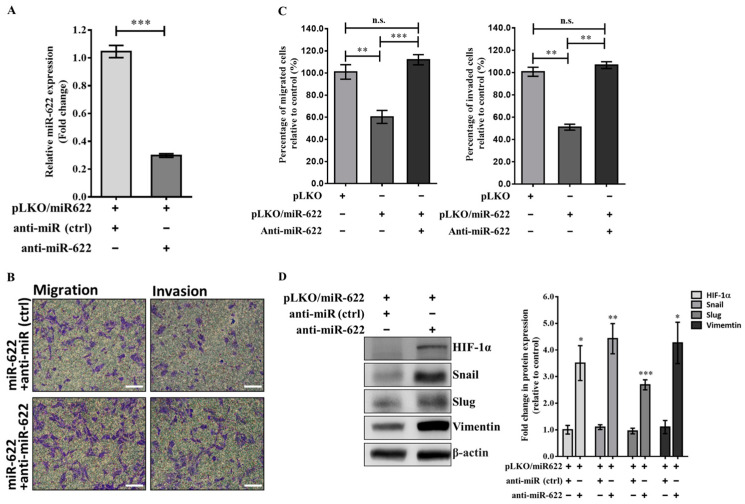
Restoring the expression of the EMT protein is achieved by inhibiting miR-622, leading to increased migration and invasion of breast cancer cells. (**A**) The expression level of the miR-622 transcript in MDA-MB-231 cells carrying pLKO/miR-622 was significantly reduced by treatment with a specific anti-miR^TM^ miRNA inhibitor for miR-622 (anti-miR-622). (**B**) The transwell assay was used to assess cell migration (**left panel**) and invasion (**right panel**) in cells transiently transfected with an miR-622 inhibitor (anti-miR-622) or negative control mimics (anti-miR, ctrl). The white scale bar indicates 50 μm. (**C**) Cell counts from five random fields of view at 100× magnification were averaged to represent the mean ± S.D. of cells per field of view, obtained from three independent experiments. n.s., non-significantand statistical significance is denoted by asterisks: * *p <* 0.05, ** *p* < 0.01 and *** *p* < 0.001. (**D**) Western blot analysis shows the levels of HIF-1α and EMT markers (Snail, Slug, and vimentin) in MDA-MB-231 breast cancer cells treated with the miR-622 inhibitor (anti-miR-622) compared to cells treated with the negative control miRNA inhibitor (anti-miR, ctrl). Quantitative analysis of Western blot results is presented as the means ± S.D. The fold change was normalized to the internal control of β-actin. * *p* < 0.05, ** *p* < 0.01, and *** *p* < 0.001. The original western blot figures can be found in File S1.

Furthermore, the substantial increase in miR-622 levels observed in miR-622-transfected MDA-MB-231 cells was effectively reduced after treatment with a specific anti-miR^TM^ miRNA inhibitor for miR-622 (anti-miR-622) (Ambion, Inc., Austin, TX, USA) (Figure 3A). A significantly higher proportion of breast cancer cells demonstrated a twofold increase in migration and invasion capabilities when pLKO/miR-622 transfectants were treated with the miR-622 inhibitor in the transwell migration assay, compared to cells transfected with the pLKO/miR-622 (Figure 3B,C). Western blot analysis revealed restoration of HIF-1α levels and significantly elevated levels of Snail, Slug, and vimentin proteins in MDA-MB-231 cells transfected with pLKO/miR-622 after treatment with an inhibitor targeting miR-622 (Figure 3D).

### 3.4. MiR-622 Upregulates miR-30a Expression in Contributing to the Suppression of Breast Cancer Cell Metastasis by Inhibiting EMT

**Figure 4:**
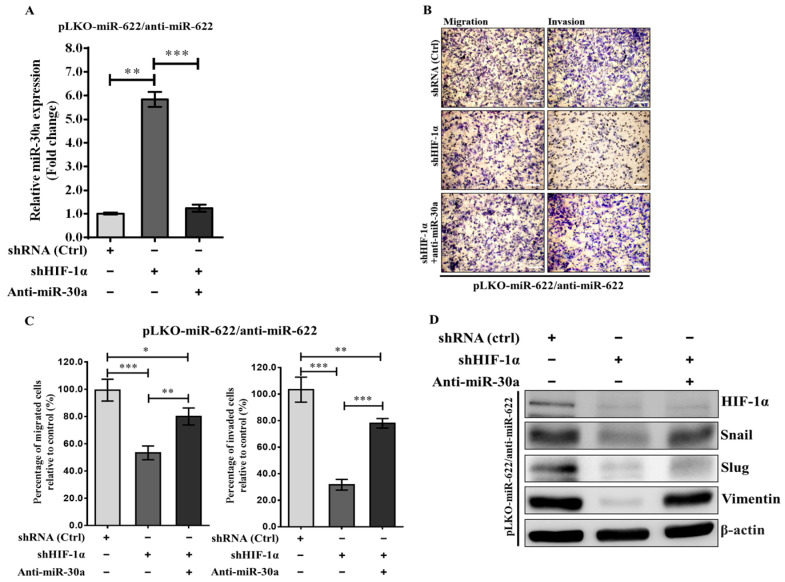
Impact of miR-622 inhibition on breast cancer cell progression through modulation of HIF-1α or miR-30a in MDA-MB-231 cells with pLKO/miR-622. (**A**) The expression level of miR-30a was quantitated in anti-miR-622-treated MDA-MBA-231/pLKO-miR-622 cells alongside HIF-1α knockdown (short-hairpin RNA against HIF-1α, shHIF-1α) or a specific inhibitor against miR-30a (anti-miR-30a). (**B**) Representative micrographs from the transwell migration assay depict crystal-violet-stained migration (**left panel**) and invasion (**right panel**) filter membranes. The white scale bar indicates 50 μm. (**C**) A significant decrease in migration and invasion abilities observed in shHIF-1α-treated cells was attenuated after cotreatment with an miR-30a inhibitor (anti-miR-30a). Quantitative analysis of migration and invasion is presented, with values that indicate the means ± S.D. from triplicate experiments. Statistical significance is denoted by asterisks: * *p* < 0.05, ** *p* < 0.01, *** *p* < 0.001. (**D**) Western blot analysis represented the protein levels of HIF-1α and mesenchymal markers in MDA-MB-231 cells transfected with pLKO/miR-622 and then subjected to various treatments, including anti-miR-622, anti-miR-30a, and shHIF-1α. The original western blot figures can be found in File S1.

In our previous studies, we identified *Slug* and *Vimentin* mRNAs as direct targets of miR-30a [12,13]. This current research underscores the potential of miR-622 to inhibit metastasis in breast cancer cells by targeting HIF-1α, counteracting mesenchymal proteins (Figure 2). Expanding on this knowledge, our current investigation explores the molecular mechanisms involving miRNA synergy, specifically the interaction between miR-622 and miR-30a, that govern the inhibitory effects on breast cancer cell metastasis. We focus on the potential role in inhibiting HIF-1α-mediated EMT through miR-30. Initially, we used short-hairpin RNA against HIF-1α (shHIF-1α) to silence HIF-1α protein expression. This intervention resulted in an approximate sixfold increase in miR-30a levels in MDA-MB-231 cells transfected with pLKO/miR-622 when coadministered with the miR-622 inhibitor. The induction of the miR-30a level was reversed when treated with the miR-30a inhibitor (anti-miR-30a) (Figure 4A). Concurrently, shHIF-1α-knockdown treatment in MDA-MB-231–pLKO/miR-622 cells administered the miR-622 inhibitor led to a significant reduction of more than 50% in migration and invasion activity compared to negative control (shRNA, ctrl) in the transwell assay (Figure 4B,C). This suppression was reversed when anti-miR-30a was added to MDA-MB-231 cells carrying pLKO/miR-622 and then treated with an miR-622 inhibitor (Figure 4C). The results of the Western blot assay showed suppression of EMT-related proteins when shHIF-1α was introduced (Figure 4D). On the contrary, elevated levels of mesenchymal markers, including Snail, Slug, and vimentin, were observed when miR-622-overexpressing MDA-MB-231 cells were treated with anti-miR-622 and sequentially cotreated with shHIF-1α and anti-miR-30a (Figure 4D). These collective findings highlight the crucial role of miR-622, demonstrated by its interaction with HIF-1α in the management of antimetastatic effects. This regulation is achieved by modulating miR-30a expression, which disrupts the EMT process in invasive breast cancer.

### 3.5. MiR-622 Represses HIF-1α to Upregulate miR-30a Transcription

**Figure 5:**
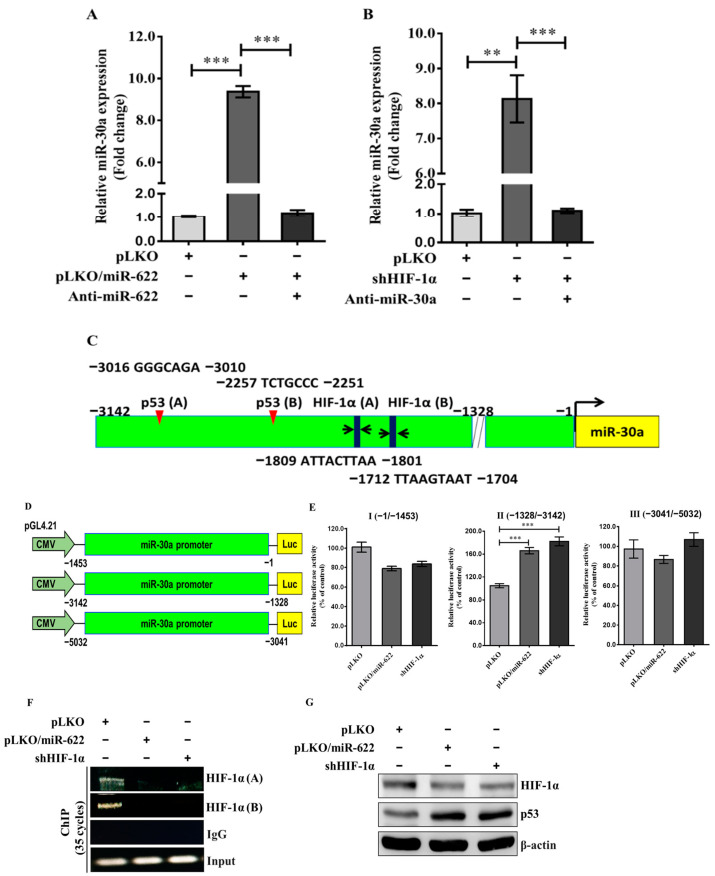
MiR-622 upregulates the level of miR-30a through the repression of HIF-1α. Quantification of miR-30a level using qRT-PCR in MDA-MB-231 cells transfected with pLKO/miR-622 after treatment with the miR-622 inhibitor (anti-miR-622) is shown in (**A**) and in (**B**) after HIF-1α knockdown (shHIF-1α) along with the miR-30a inhibitor (anti-miR-30a) in MDA-MB-231 cells. *RNU6B* served as an internal control. (**C**) Schematic representation of miR-30a promoter regions inserted upstream of the pGL4.21 vector. (**D**) Schematic overview of various luciferase reporter constructs from the miR-30a promoter region and the results of the promoter activity assay performed in MDA-MB-231 cells transfected with pLKO/miR-622 or subjected to HIF-1α-knockdown treatment (shHIF-1α). The data histograms and error bars indicate the means ± S.D. of the independent samples in triplicate. Statistical significance is denoted by asterisks: ** *p* < 0.01 and *** *p* < 0.001. (**E**) Representation of nucleotide sequences that encompass two HIF-1α- and p53-binding motifs within the promoter region (nt −1328~−3142) of miR-30a. (**F**) Identification of two binding motifs to the HIF-1α of the miR-30a promoter region was performed using a chromatin immunoprecipitation (ChIP) assay against an HIF-1α-specific antibody. IgG served as a negative control. (**G**) Representative Western blot indicates HIF-1α and p53 proteins in cells treated with pLKO/miR-622 or shHIF-1α with β-actin utilized as internal control. The original western blot figures can be found in File S1.

As noted previously, inhibition of HIF-1α leads to an increase in miR-30a, which disrupts the process and subsequently reduces the metastatic potential of breast cancer cells. We sought to investigate whether the decrease in miR-30a caused by the overexpression of HIF-1α could be attributed to the low level of miR-622. Our hypothesis was supported by the observation that MDA-MB-231 cells transfected with pLKO/miR-622 exhibited significantly higher levels of miR-30a compared to the control group (Figure 5A); this finding reflects the effects observed with shHIF-1α treatment (Figure 5B). Furthermore, an increase in miR-30a level was closely associated with miR-622 induction, paralleling the effects of shHIF-1α treatment. In contrast, the previous increase in miR-30a was effectively reversed by treatment with anti-miR-622 (Figure 5A) or anti-miR-30a (Figure 5B). These findings provide strong support for our hypothesis that MDA-MB-231/pLKO-miR-622 cells elevated miR-30a levels by repressing HIF-1α.

Furthermore, we explored the primary miRNA transcript of miR-30a (pri-miR-30a), located on chromosome 6q13. Through bioinformatic analysis, ALLGGEN_PROMO predicted two potential HIF-1α- and two p53-binding sites in the promoter region (Figure S2) preceding the transcriptional start site of pri-miR-30a (Figure 5C). Our proposed mechanism for upregulating miR-30a through p53 likely involves downregulation of HIF-1α. To investigate this, we carried out an experiment involving HIF-1α knockdown along with modified promoter constructs of pri-miR-30a. Three fragments upstream of the 5′-UTR of the pri-miR-30a gene were integrated into the pGL4.12-Basic-Luc vector, designated as (−1453/−1)-Luc, (−3142/−1328)-Luc, and (−5032/−3041)-Luc (Figure 5D). In the presence of pLKO/miR-622 or shHIF-1α, MDA-MB-231 cells carrying the promoter construct (−3142/−1328)-Luc exhibited an approximate twofold increase in luciferase activity compared to the negative control (Figure 5E). Moreover, chromatin immunoprecipitation assays conducted with an antibody against HIF-1α revealed that HIF-1α protein binds to the upstream promoter region between −3142 and −1328 of the pri-miR-30a gene (Figure 5F). Furthermore, an elevated level of p53 correlates inversely with a reduction in HIF-1α level in MDA-MB-231 cells carrying pLKO/miR-622 or treated with shHIF-1α compared to the control group (Figure 5G). Collectively, these findings indicate that miR-622 plays a crucial role in promoting miR-30a production, thus reversing breast cancer cell metastasis through suppression of HIF-1α expression.

### 3.6. MiR-622 Reverses the Docetaxel Sensitivity of Breast Cancer Cells

**Figure 6:**
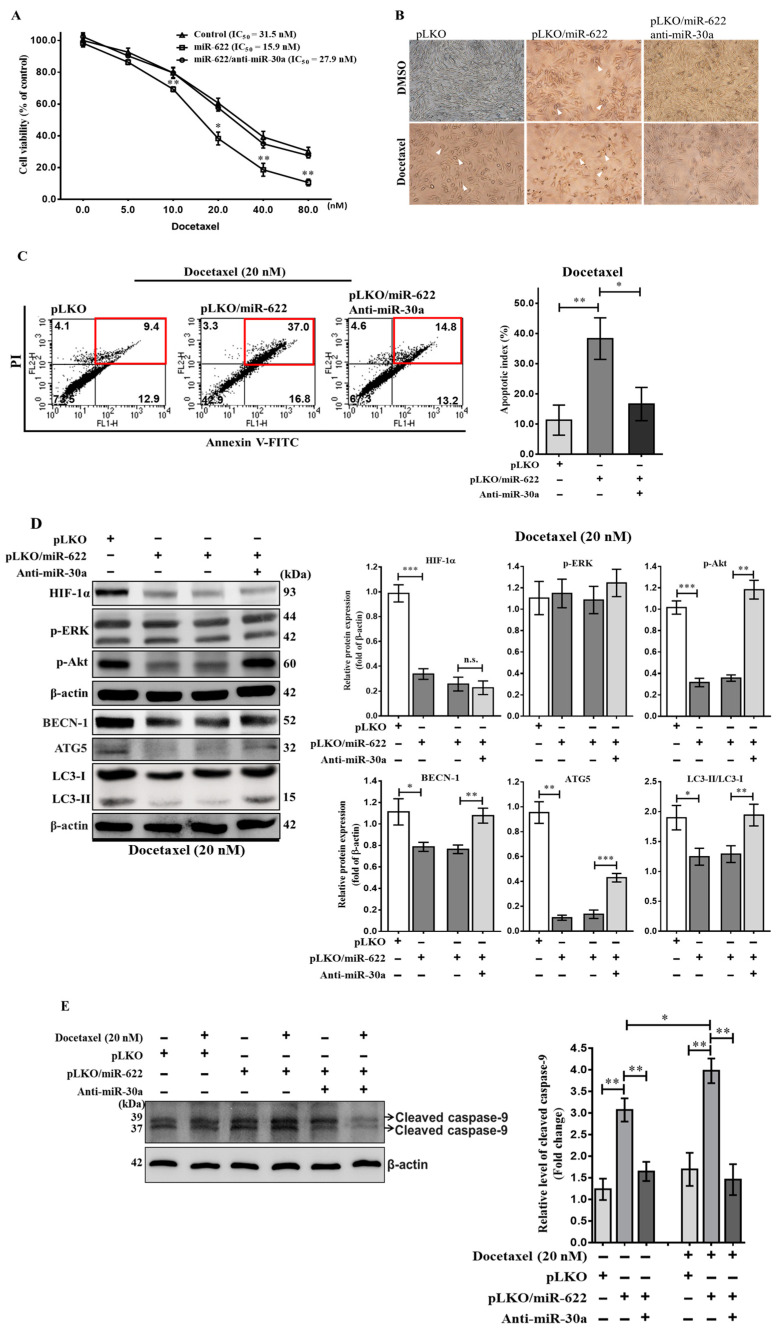
Restoration of docetaxel sensitivity in breast cancer cells through miR-622-induced expression of miR-30a. (**A**) Evaluation of the impact of miR-622 on MDA-MB-231 cell viability after a 48 h treatment with varying concentrations of docetaxel, as indicated, using the MTT assay. (**B**) Representative images of cells exposed to docetaxel (20 nM) or carrying pLKO/miR-622 captured using an inverted phase contrast microscope at a magnification of 200×. The white arrow indicates a shrunken morphology with rounded cell bodies. (**C**) Apoptosis assay using flow cytometry after staining with annexin V–FITC/propidium iodide (PI). Representative scatterplots of PI (*y*-axis) versus annexin V (*x*-axis). Apoptotic cells are indicated by a red frame. Data are presented as apoptotic cells based on independent experiments performed in triplicate and analyzed by FACS. (**D**) Representative images of Western blot analysis showing HIF-1α- and autophagy-related proteins and (**E**) caspase 9 activation (cleaved caspase 9) under 20 nM docetaxel in conjunction with various treatments as indicated. The densitometric analysis of the protein bands was performed using Digital Protein Imagineware, with β-actin serving as an internal control. Data are presented as the means ± S.D. of three independent experiments. n.s., non-significant and statistical significance is indicated as * *p* < 0.05, ** *p* < 0.01, *** *p* < 0.001. The original western blot figures can be found in File S1.

In addition to its role in inhibiting cancer cell metastasis, miR-30a has recently received attention for its potential to sensitize drug-resistant lung cancer cells by impeding beclin 1-executed autophagy [20]. Therefore, we evaluated the impact of beclin 1-mediated autophagy on reduced drug sensitivity in breast cancer chemoresistance attributed to the reduction in miR-30a caused by HIF-1α through the loss of miR-622. Using the MTT assay with various concentrations of docetaxel for 48 h, MDA-MB-231 breast cancer cells carrying pLKO/miR-622 exhibited increased docetaxel sensitivity, as evidenced by a lower IC_50_ (15.9 ± 1.0 nM) compared to the control group (31.5 ± 1.1 nM). However, the administration of MDA-MB-231-pLKO/miR-622 cells with anti-miR-30a had the opposite effect (27.9 ± 1.2 nM) (Figure 6A). Cells exposed to docetaxel (20 nM) or carrying pLKO/miR-622 displayed a shrunken morphology with rounded cells, observed under an inverted phase contrast microscope (Figure 6B). Flow cytometric assays were used to assess cell apoptosis. In the presence of 20 nM docetaxel, there was an approximate fourfold increase in apoptosis rate in MDA-MB-231-pLKO/miR-622 cells (38.3%) compared to the control group (11.4%) (Figure 6C). However, the introduction of anti-miR-30a into MDA-MB-231-pLKO/miR-622 cells resulted in a dramatic 60% reduction in the apoptosis rate (16.6%) (Figure 6C).

To understand docetaxel resistance in MDA-MB-231-pLKO/miR-622 cells by inhibiting miR-30a, we evaluated the impact of anti-miR-30a treatment on beclin 1-mediated autophagy signaling in breast cancer cells transfected with pLKO/miR-622. As expected, miR-30a reduced ATG5 and the activated form of LC3-II, correlated with decreased HIF-1α level in MDA-MB-231-pLKO/miR-622 cells, as observed in Western blot analysis (Figure 6D). On the contrary, anti-miR-30a treatment in MDA-MB-231-pLKO/miR-622 cells reversed the decrease in activated LC3-II expression (Figure 6D). We also evaluated autophagy induction via phosphorylation of the ERK (p-ERK) or Akt (p-Akt) protein, known contributors to drug resistance in cancers [21,22]. In Figure 6D, the level of p-Akt decreased in MDA-MB-231 cells transfected with pLKO/miR-622, leading to cell death, while a significant increase in the level of p-Akt reversed the effect on autophagy induction when treated with anti-miR-30a in breast cancer cells overexpressing miR-622. Furthermore, Western blot analysis revealed enhanced caspase 9 cleavage due to miR-622 upregulated (Figure 6E). The apoptotic effect caused by miR-622 that is linked to activation of cleaved caspase 9 was significantly reduced after anti-miR-30a treatment. In summary, miR-30a played a role in the in vitro chemoresistance of breast cancer cells, partially through interactions between miR-622 and miR-30a, suppressing p-Akt-mediated autophagy signaling.

## 4. Discussion

**Figure 7:**
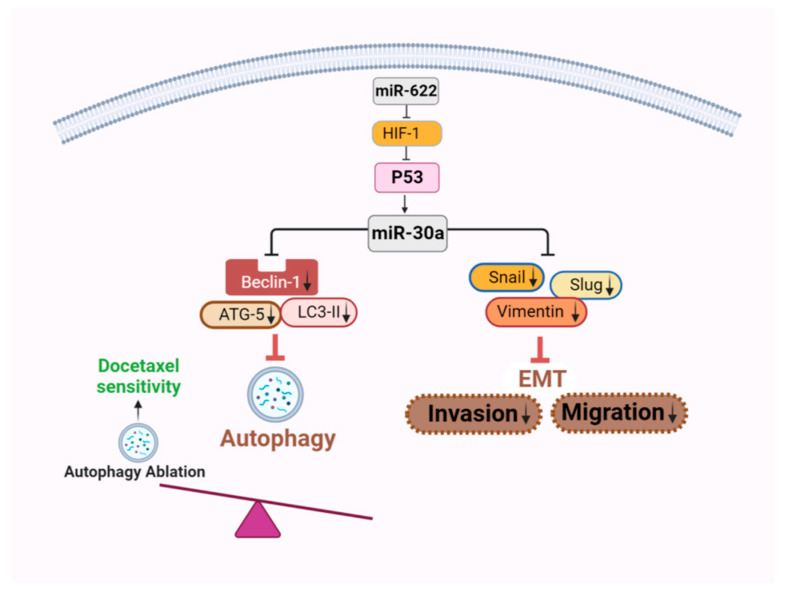
A schematic representation elucidating the mechanism by which miR-622-induced miR-30a expression influences EMT and autophagy signaling interference. The overexpression of miR-622 results in downregulation of HIF-1α, subsequently inducing miR-30a expression. This induction in turn hinders the activation of EMT and autophagy signaling. Consequently, this inhibitory effect leads to the suppression of invasion and migration, along with the restoration of drug sensitivity in breast cancer cells. The downward arrows indicate a decrease in expression levels. This illustrative diagram was created using BioRender scientific illustration software (version 1.0), available on the website https://www.biorender.com/ (accessed on 13 December 2023).

Metastatic cancer is the primary cause of death in up to 90% of cancer patients [23]. Metastasis, often driven by the EMT program and occurring in hypoxic environments, underscores the complexity of cancer progression. In particular, studies have emphasized the role of miRNAs in modulating oncogenes and tumor cell progression. Our investigation observed significantly lower levels of miR-622 in highly invasive Hs578T and MDA-MB-231 cell lines compared to the benign MCF-7 cell line in breast cancer. Previous research, including our own, delineates the inhibitory roles of miR-622 in cancer cell metastasis across various types of cancer [15,18,19], including breast cancer [24]. We acknowledge the significance of miR-30a and its combined effects with the suppression of HIF-1α within our experimental framework. We stress the extensively documented mechanism by which miR-30a suppresses invasion and metastasis of breast cancer cells and inactivates autophagy [25,26], as demonstrated in our previous studies [12,13]. In this investigation, the introduction of miR-622 counteracted malignant metastasis in vitro by inhibiting HIF-1α-related EMT signaling and increasing miR-30a levels. This augmentation improved docetaxel sensitivity in TNBC cells by suppressing autophagy, supported by miR-622. Our findings underscore the biological importance of miRNA interactions, particularly highlighting the roles of miR-622 and miR-30a in drug sensitivity and in mitigating adverse outcomes in breast cancer (Figure 7). Our ongoing research aims to dive into the synergistic effects of miR-622 and miR-30a, expanding on these established findings. Beyond the individual effects of miRNAs, we seek to unveil the novel synergy between miR-622 and miR-30a, representing unexplored territory in the field. This collaborative action represents the distinguishing feature of our current investigation, offering new insights into breast cancer metastasis and treatment strategies.

Hypoxia-induced EMT emerges as a critical mechanism that forces cancer cells to disseminate from primary sites and establish new clones. Tumor cell expansion, coupled with HIF-1α overexpression, promotes EMT, triggering loss of cell-to-cell adhesion through upregulation of EMT transcription factors, including Snail, Slug, Twist, and ZEB1 [27]. Clinical samples obtained from breast cancer patients who exhibited poor outcomes, including advanced stages, lymph node metastases, and decreased disease-free survival rates, underscore a direct relationship between increased vimentin and Slug expression and decreased miR-30a levels [12,13]. In the present investigation, our primary objective was to clarify the role of miR-622 in impeding the invasive and metastatic characteristics of breast cancer cells using in vitro cell models. We acknowledge the inherent disparity between in vitro and in vivo studies, especially with regard to the elucidation of mechanisms hindering HIF-1α-mediated EMT signaling. This underscores the tumor-suppressive function of miR-622 in our cell-based assays. Our prior research mainly concentrated on lung cancer, using a mouse xenotransplantation model to validate the ability of miR-622 to inhibit tumor growth and suppress lung tumor metastases after tail vein injection [15]. Although this investigation was not directly related to breast cancer, the results obtained offer partial support for the potential of miR-622 to mitigate the invasion and migration capabilities of breast cancer cells, drawing insights from our lung cancer study.

We conducted HIF-1α-knockdown experiments using shRNA-HIF-1α or miR-622, which revealed HIF-1α’s binding to the miR-30a promoter, thus initiating epigenetic regulation that suppresses miR-30a transcription and contributes to the observed clinical signature. Downregulation of HIF-1α by miR-622, which deactivates genes within the EMT pathway, culminates in decreased levels of Snail, Slug, and vimentin proteins through miR-30a, consequently attenuating the invasion and migration capacities of breast cancer cells. These findings underscore the potential of miR-622 as a predictive marker for inhibiting breast cancer invasiveness. Recent discoveries have illuminated additional aspects of HIF-1α’s regulatory roles. Specifically, HIF-1α interacts with hormone response elements (HREs) within the promoter region of lncRNA BC005927, inducing the oncogenic role of lncRNA BC005927 in gastric cancer through the upregulation of the EPH receptor B4 (EPHB4) [28]. Moreover, HIF-1α recruits histone deacetylase 1 (HDAC1) to the promoter of the pri-miR-548an gene, leading to transcriptional suppression of miR-548an expression. This upregulation of EMT markers facilitates the proliferation and invasion of pancreatic cancer cells [29]. These insights into the regulation of non-coding RNAs by hypoxia signaling present promising avenues for the development of therapeutic interventions against cancer progression.

Cellular autophagy is indispensable to maintain homeostasis and ensure cell survival, exerting a nuanced influence on cancer. While it can mitigate the onset of cancer in certain cases, it may also improve the survival of cancer stem cells, facilitate EMT, and increase tumor aggressiveness within their environment [30]. In particular, in the hypoxic environment characteristic of human solid tumors, both the beclin 1 protein and mRNA exhibit elevation alongside HIF-1α [31,32,33,34]. Studies suggest that autophagy’s impact on tumor cell progression involves suppression of autophagy signaling genes through miRNAs, thus improving responses to anticancer agents [35]. Our investigation illustrates that inhibiting miR-30a using an antagomir molecule increases the levels of ATG5 protein to activate LC3-II, which in turn reduces apoptosis and then promotes resistance to docetaxel in MDA-MB-231 cells. These findings are consistent with the results observed in other studies on chemotherapy-resistant cancer cells [20,36,37]. Furthermore, HIF-1α-induced activation of autophagy assumes a pivotal role in liver and bladder cancer cells [9,38]. The initiation of autophagy can be driven by the activation of p-ERK and p-Akt, recognized contributors to drug resistance in cancers [18,19]. However, in our replicated experiments, we did not discern significant alterations in p-ERK expression levels in MDA-MB-231 cells carrying pLKO-622 compared to the control group. Intriguingly, the existing literature posits that p-ERK might participate in the promotion of HIF-1α transcription in cancer cells under hypoxic conditions [39]. The absence of pronounced changes in p-ERK levels in our study underscores the intricate interplay between signaling pathways regulating HIF-1α expression within our experimental setup. This observation hints at the possibility that subtle fluctuations in p-ERK levels could underscore its pivotal role as a key regulator of HIF-1 production and its downstream targets. Understanding this relationship could facilitate deciphering the complex mechanism linking ERK phosphorylation and HIF-1 production, crucial for cancer cell progression and survival under hypoxic conditions. Therefore, the development of specific inhibitors to attenuate advanced autophagy processes mediated by ERK and Akt, consequently quelling the expression of autophagy-related genes, presents a promising avenue for combating drug-resistant cancers influenced by HIF-1α-induced autophagy. The translation of these findings from laboratory settings into in vivo models holds substantial promise for therapeutic interventions. Substantiating these results through in vivo studies would further enhance the consideration of miR-622 as a viable therapeutic target in mitigating breast cancer progression. Thus, incorporation of in vivo experiments would significantly increase our understanding of the clinical applicability and potential of miR-622 in future drug development for breast cancer treatment.

In this study, our data corroborate the role of miR-622 in facilitating miR-30a transcription, consequently counteracting breast cancer cell metastasis through suppression of HIF-1α expression. It is important to note that the luciferase activity depicted in the promoter construct (−1453/−1)-Luc appears modest, although not statistically significant (Figure 5E). We recognize that this observation may prompt inquiries. Our conjecture is that MDA-MB-231 cells that carry miR-622 could impede other downstream transcription factors, potentially influencing the HIF-1α binding to the transcriptional start site of pri-miR-30a. Such modulation could conceivably affect the transcriptional expression of miR-30a. Further experiments are imperative to elucidate a comprehensive understanding of the intricate regulatory mechanisms that might involve them. These additional experiments are intended to offer a clearer insight into the observed modest luciferase activity and its underlying regulatory mechanisms. The objective is to delineate the potential influence of miR-622 on the transcriptional regulation of miR-30a and its subsequent ramifications on HIF-1α binding. This effort is crucial to comprehensively elucidate the regulatory dynamics at play and to advance our understanding of the interaction between miR-622, miR-30a, and HIF-1α in breast cancer metastasis.

Recent investigations suggest that miR-30a downregulation is correlated with p53 inactivation, associating it with lymph node metastasis and unfavorable prognoses in TNBC [40]. A parallel correlation between reduced miR-30a expression and p53 was observed in studies of non-small-cell lung cancer [41]. Situated on chromosome 6q13, miR-30a plays a role in restraining the aggressiveness of breast cancer cells, with loss of heterozygosity on chromosome 6q13 indicating its suppressive function [42]. Additionally, p53 binds directly to the miR-30a promoter and its inactivation leads to miR-30a downregulation, fostering invasive breast cancer phenotypes [40]. Emerging evidence suggests that p53 serves as an autophagy inhibitor, interrupting autophagic homeostasis and tumorigenesis [43]. HIF-1α links p53 deficiency with hypoxia-mediated signaling, promoting cancer aggressiveness, while suppression of p53 can increase autophagy in tumor progression [44] and counteracts p53-mediated apoptosis [45]. Our study demonstrates that miR-622 can modulate the expression levels of beclin 1-dependent genes, altering cancer progression and reinstating drug sensitivity by suppressing HIF-1α expression in breast cancer cells. This discovery unveils a novel synergy between miR-622 and miR-30a in the regulation of autophagy through p53 activation. Although autophagy activation associated with p53 status is considered crucial in cancer therapy [46,47], the molecular mechanisms underlying the inhibition of tumor autophagy due to miR-30a elevation, which improves drug sensitivity, are explored as potent suppressors of cancer cell progression. Moreover, understanding the tissue- or organ-specific nuances of the interaction between miR-30a and p53 in the regulation of autophagy in different types of cancers is essential for effective cancer management. Thorough investigation of tumor miRNA biology to attenuate autophagy in hypoxic environments may illustrate the role of non-coding RNA molecules in improving the invasiveness of breast cancer.

## 5. Conclusions

In summary, our findings elucidate that upregulation of miR-622 and the consequent transcription of miR-30a modulate autophagy, presenting novel therapeutic pathways to enhance chemosensitivity, reverse EMT, mitigate invasion and migration, and impede autophagy in cancer biology. These insights may pave the way for innovative therapeutic strategies to address aggressive breast cancer.

## Data Availability

All data presented in this study are available on request from the corresponding author.

## Supplementary Materials

The following supporting information can be downloaded at https://www.mdpi.com/article/10.3390/cancers16030657/s1, Figure S1: HIF-1α gene as a direct target of miR-622; Figure S2: HIF-α-binding sites in the pri-miR-30a promoter region; File S1: Full pictures of the Western blots.
