# Supplementary material for: miR-622 Increases miR-30a Expression through Inhibition of Hypoxia-Inducible Factor 1α to Improve Metastasis and Chemoresistance in Human Invasive Breast Cancer Cells
Source: Cancers (Basel). 2024 Feb 3;16(3):657. doi: 10.3390/cancers16030657 (PMC10854867; doi:10.3390/cancers16030657)
Supplement: Supplementary file 1 [file cancers-16-00657-s001.zip › cancers-2856959-File S1..pdf]

Western blots of Figure 1B

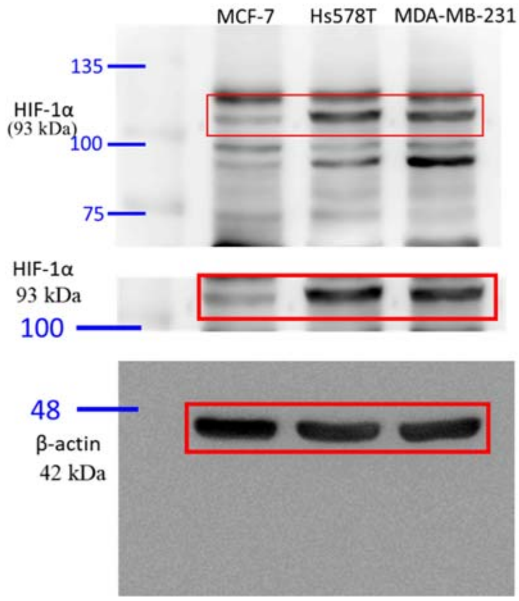

Blots probed with anti-HIF-1α (Proteintech, no. 66730-1-Ig; 93 kDa)

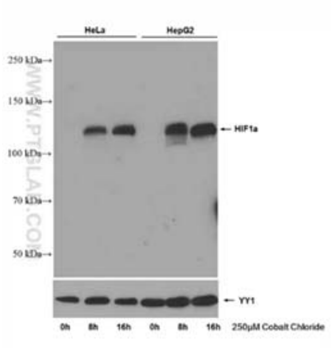

Anti-β-actin (Novus, AC-15; 42 kDa)

Western blots of Figure 2D

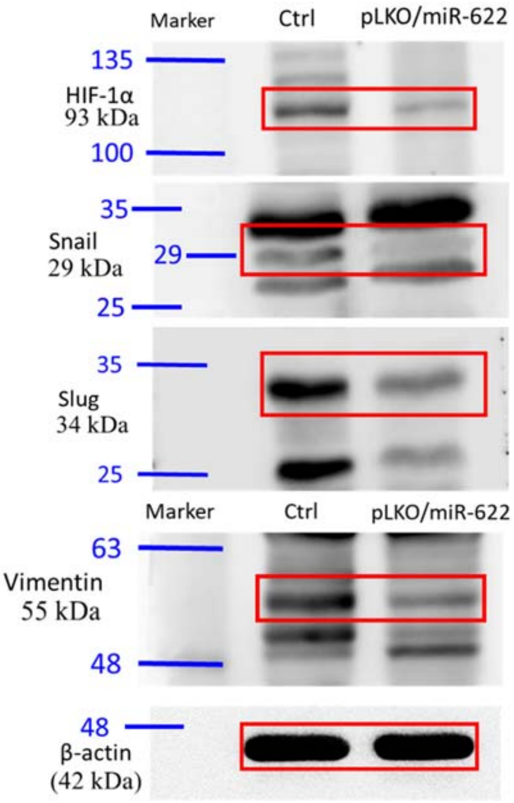

Anti-HIF-1α (Proteintech, no. 66730-1-Ig; 93 kDa),
Anti-Snail (Santa Cruz, no.10432; 29 kDa),
Anti-Slug (Cell Signaling, no. 9585; 34 kDa),
Anti-Vimentin (Cell Signaling, no. 5741; 55 kDa),
Anti-β-actin (Novus, AC-15; 42 kDa).

Western blots of Figure 3D

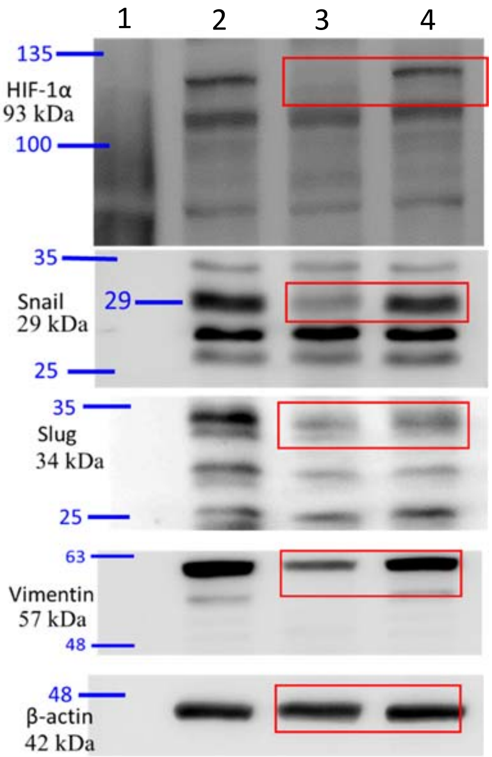

Lane 1. marker
Lane 2. pLKO (crtl)
Lane 3. pLKO/miR-622
Lane 4. pLKO/miR-622 and miR-622 inhibitor

Anti-HIF-1α (Proteintech, no. 66730-1-Ig; 93 kDa),
Anti-Snail (GeneTex, no. 100754; 41 kDa),
Anti-Slug (Cell Signaling, no. 9585; 34 kDa),
Anti-Vimentin (Cell Signaling, no. 5741; 55 kDa),
Anti-β-actin (Novus, AC-15; 42 kDa).

Western blots of Figure 4D

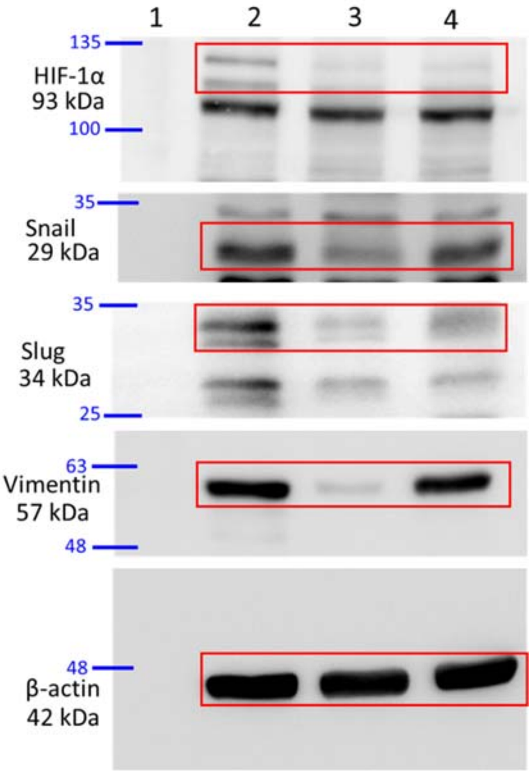

pLKO/miR-622 and miR-622 inhibitor
Lane 1. marker
Lane 2. shRNA (ctrl)
Lane 3. shHIF-1α
Lane 4. shHIF-1α and anti-miR-30a

Anti-HIF-1α (Proteintech, no. 66730-1-Ig; 93 kDa),
Anti-Snail (GeneTex, no. 100754; 41 kDa),
Anti-Slug (Cell Signaling, no. 9585; 34 kDa),
Anti-Vimentin (Cell Signaling, no. 5741; 55 kDa),
Anti-β-actin (Novus, AC-15; 42 kDa).

Western blots of Figure 5G

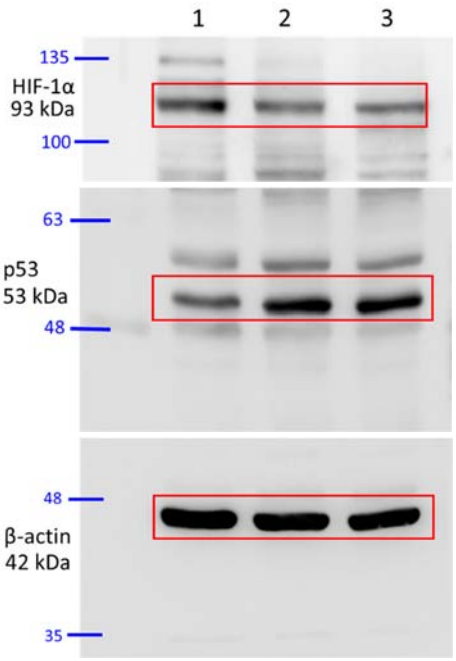

Lane 1. pLKO (ctrl)
Lane 2. pLKO/miR-622
Lane 3. shHIF-1α

Anti-HIF-1α (Proteintech, no. 66730-1-Ig; 93 kDa),
Anti-p53 (GeneTex, no. 128135; 53 kDa),
Anti-β-actin (Novus, AC-15; 42 kDa).

Western blots of Figure 6D

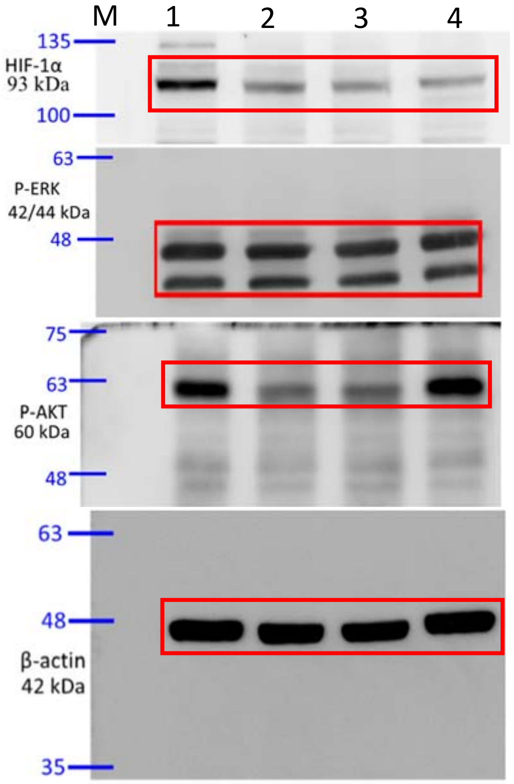

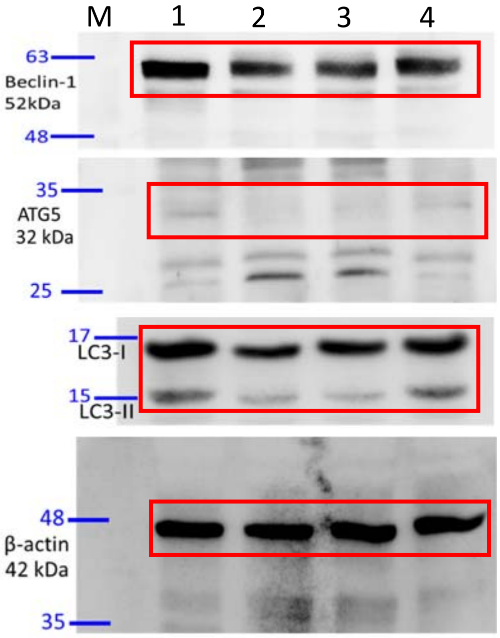

M: Marker
Lane 1: pLKO (ctrl)
Lane 2: pLKO/miR-622
Lane 3: pLKO/miR-622
Lane 4: pLKO/miR-622/miR-30a inhibitor

Anti-HIF-1α (Proteintech, no. 66730-1-Ig; 93 kDa),
Anti-p-ERK (Santa cruz, no. 7383; 41 kDa),
Anti-p-AKT (Santa cruz, no. 16646-R; 34 kDa),
Anti-Beclin-1 (Proteintech, no. 11306-1-AP; 52 kDa),
Anti-ATG5 (GeneTex, no. 31372; 32 kDa),
Anti-LC3B (GeneTex, no. 127375; 15 kDa),
Anti-β-actin (Novus, AC-15; 42 kDa).

Western blots of Figure 6E

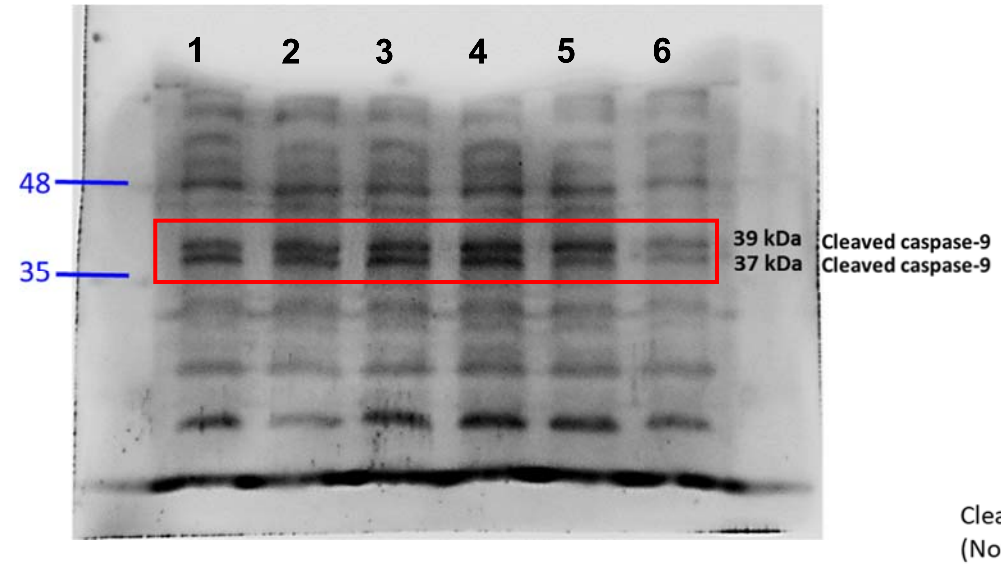

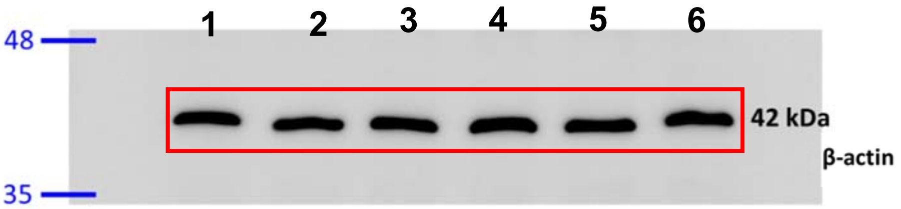

Cleaved caspase-9
(Novus, no. NB100-56118; 37 KDa),
Anti-β-actin (Novus, AC-15; 42 KDa).

Lane 1: pLKO (ctrl)

Lane 2: pLKO (ctrl)/docetaxel

Lane 3: pLKO/miR-622

Lane 4: pLKO/miR-622/docetaxel

Lane 5: pLKO/miR-622/anti-miR-30a

Lane 6: pLKO/miR-622/anti-miR-30a/docetaxel
