# Supplementary material for: miR-622 Increases miR-30a Expression through Inhibition of Hypoxia-Inducible Factor 1α to Improve Metastasis and Chemoresistance in Human Invasive Breast Cancer Cells
Source: Cancers (Basel). 2024 Feb 3;16(3):657. doi: 10.3390/cancers16030657 (PMC10854867; doi:10.3390/cancers16030657)
Supplement: Supplementary file 1 [file cancers-16-00657-s001.zip › cancers-2856959-supplementary.pdf]

A.

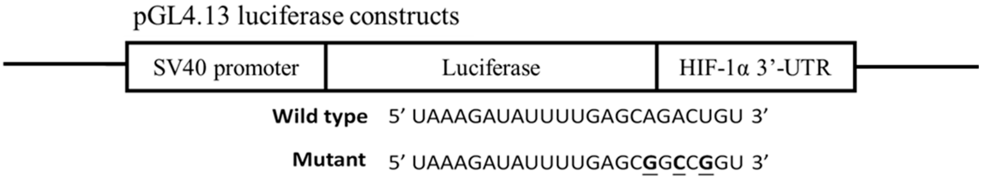

B.

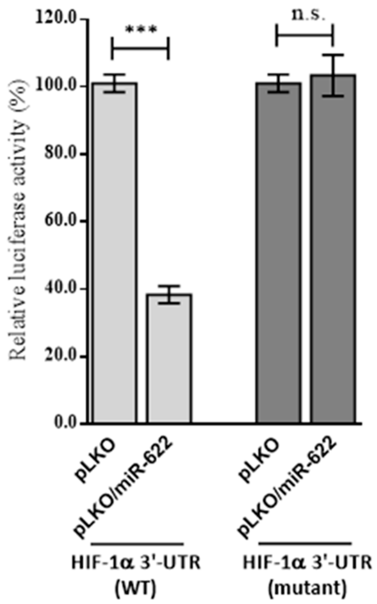

Figure S1: HIF-1α gene as a direct target of miR-622. (A) The diagram illustrates the luciferase reporter constructs. The three highlighted nucleotides indicate mutated sites in the HIF-1α mRNA, created through site-directed mutagenesis to result in mismatches with the complementary sequence of miR-622. (B) MDA-MB-231 cells were cultured in 24-well plates and transfected with 100 ng of either wild-type or mutated HIF-1α 3’-UTR constructs, in addition to pLKO/miR-622. The firefly luciferase/Renilla luciferase activity ratio of each sample was determined using a dual-luciferase reporter assay system. Each bar represents the mean ± S.D. of results from three independent experiments; *** indicates a significance level of $p < 0.001$.

841 ATGGTTCTCT GCCCTGAAGG TGTAGCAAAA TTGTTTGATA GCTTTTGCAA AATTTACATA
901 CCCAGCAGCC TCCCCATCAG CTGAATAAGA ATTGCAATGT GGATGTGGCA GAGGCAATCA
961 GGTATGTGGG GTTAGAAGAA GCTGTCCTGG TCATTCTGTT GCCACCCTCT CACTTGGAGA
1021 ATTGCTGATCCAATGAATTC CTTAACATAA CTTTGAAAAC ACAGCGTTTT GCTCAGAACT
1081 TCATGAATAA ACATCAAATT TTCACTAAAC AAAATAATTA AAGACCATAA GATCTTAGTT
1141 ATAATACTTC ATAAATGGAA AAGCACTTTA TTGCCTGTAT GTATGTGGGC TCCTCTTTTA
1201 GCAAGATATG AGCTAGGTGA AGTCTATGTG AAAGCTAAAG CAGATTTATTCAGT*CAGAT*
1261 *GCCAGACAAT TTTACCAG*TT TAACTGAGAC TCATT**ATTACTTAA**ACTACA TGCTTGTTTT

Forward primer HIF-1α (A)

1321 TTTGGTTACT GAGTAGTATA GGTCCCACTT GGATCAGCAA TTCTCAAAGT GAGAGGGTGG
1381 CAATAGAATT AT**TTAAGTAA**TTATTTGTTA TTT*GAGGTTG TTTGCTTAGT ATTTAC*TTGT

HIF-1α (B) Reverse primer

1441 CACTGTTTTA CATGTTATTT AATATATTAC AGTTATTTAA AAGTGGTAAG ATTCCAAGGC
1501 AAGTAGGGGA CCTGTCACTT TGAGAAAAAT TTATGGTGGT AAAAATATTT TCTTTTTGTA
1561 AAGAGTCACT GATATACAAT GTCCCCCCAG GGTGTAAGCC TTGGTATTTA TCTATTTTAA
1621 TTAAAGAGTA CTCTGCCTGT CTAGTCGTGG CTAACTTAAA AGAAGTGGGT ATCTTGGTGA
1681 GAAGTTCACA AGGTTGGTAG CCATAGCTGA GTTTGGTGGA TTGACTCAAG CTCTAGTCAG

Figure S2. HIF-1α binding sites in the pri-miR-30a promoter region. A schematic illustration showcases the ChIP-seq binding motif of HIF-1α within the promoter region of the primary miRNA transcript of miR-30 (pri-miR-30a), retrieved from the NCBI website (https://www.ncbi.nlm.nih.gov/nuccore/6522967). The DNA sequences for the primer set targeting the HIF-1α-binding regions (highlighted in yellow) are included. The resulting PCR fragments are 181 base pairs (bps) in length and encompass the HIF-1α binding site.
